# Two New Phenolic Glycosides from *Gnaphalium affine* D. Don and Their Anti-Complementary Activity

**DOI:** 10.3390/molecules18077751

**Published:** 2013-07-03

**Authors:** Junli Li, Doudou Huang, Wansheng Chen, Zhongxin Xi, Cheng Chen, Guanghui Huang, Lianna Sun

**Affiliations:** 1 School of Pharmacy, Second Military Medical University, 325 Guohe Road, Shanghai 200433, China; 2 Department of Pharmacy, Fujian University of Traditional Chinese Medicine, 1 Hutuo Road, Fuzhou 350108, China; 3 Department of Pharmacy, Changzheng Hospital, Second Military Medical University, 415 Fengyang Road, Shanghai 200003, China

**Keywords:** *Gnaphalium affine*, phenolic glycosides, anti-complementary activity

## Abstract

Two new phenolic glycosides, named gnaphaffine A and B (compounds **1** and **2**), were isolated from *Gnaphalium affine*. together with six known compounds, including caffeic acid (**3**), everlastoside L (**4**), isorhamnetin-7-*O*-β-d-glucopyranoside (**5**), quercetin-3-*O*-β-d-glucopyranoside (**6**), scutellarein-7-*O*-β-d-glucoside (**7**) and api-genin-7-*O*-β-d-glucopyranoside (**8**). Their structures were elucidated by spectroscopic methods, including ESI-MS and 2D NMR spectroscopy (HMQC and HMBC). All compounds were evaluated for their anti-complementary activity on the classical pathway of the complement system *in vitro*.

## 1. Introduction

**Figure 1:**
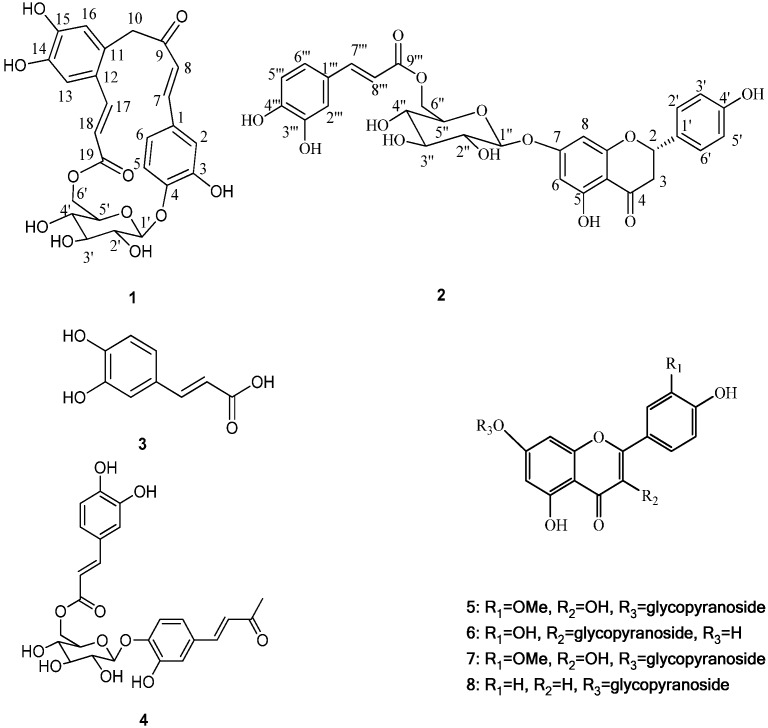
Structures of compounds **1**–**8**.

The genus *Gnaphalium* belongs to the family Compositae and consists of approximately 200 species found all over the World, among which 19 are distributed in China [1]. *Gnaphalium affine* D. Don is an annual herbaceous plant, locally named Ching Ming vegetable in China. *G. affine* has been used as a traditional medicine for the relief of swelling, wounds, lumbago, angina ache in some Latin American countries [2]. Previous phytochemical investigation on *Gnaphalium affine* have reported the isolation of flavonoids [3,4,5,6,7,8], phenolic constituents [9], polysaccharides [10], essential oil compounds [11], diterpenes [12,13], and other chemical constituents [14]. Many of these components have been demonstrated to possess anti-complementary and antifeedant activities [2]. During our ongoing research to discover novel or bioactive constituents from *G. affine*, two novel phenolic glycosides were isolated from this plant, and named gnaphalium A (**1**) and B (**2**) (Figure 1).

**Scheme 1:**
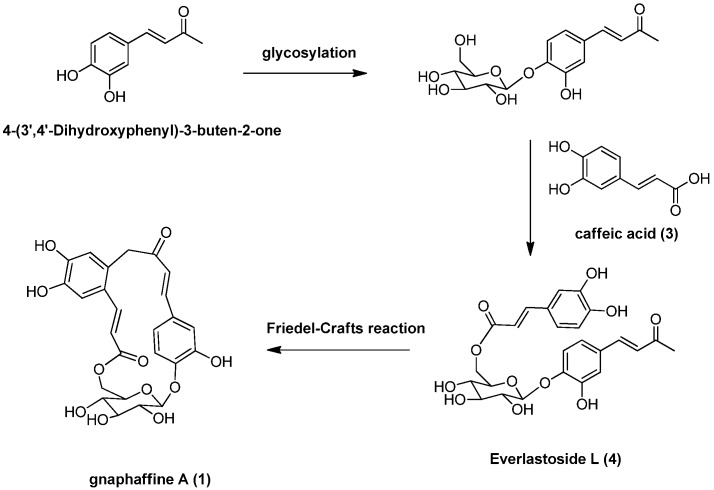
Plausible biogenetic pathway for compound **1**.

Besides, several known compounds were isolated and identified as caffeic acid (**3**) [15], everlastoside L (**4**) [16], isorhamnetin-7-*O*-β-d-glucopyranoside (**5**) [17], quercetin-3-*O*-β-d-gluco-pyranoside (**6**) [18], scutellarein-7-*O*-β-d-glucopyranoside (**7**) [19] and apigenin-7-*O*-β-d-gluco-pyranoside (**8**) [20], by comparison of their spectroscopic data with published values. Herein the isolation, characterization, and anti-complementary activity of these compounds were reported. In addition, a plausible biogenetic pathway for compound **1** has been proposed (Scheme 1).

## 2. Results and Discussion

An 80% ethanolic extract of dried *G. affine* whole plant was suspended in distilled water and extracted with EtOAc. The EtOAc soluble fraction was concentrated under reduced pressure to produce a residue that was subjected multiple chromatography, two new compounds **1** and **2** and six known compounds **3**–**8** were isolated and identified.

**Figure 2:**
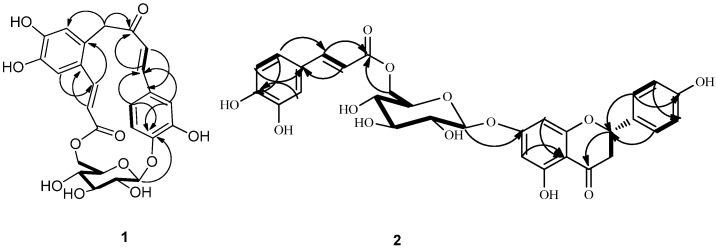
Key ^1^H-^1^H COSY (bold lines) and HMBC (H→C) correlations of compounds **1** and **2**.

Gnaphaffine A (**1**) was isolated as a yellow, amorphous powder. On the basis of a HR-ESI-MS peak at *m/z* 501.1393 [M+H]^+^ and ^13^C-NMR data the molecular formula of **1** was determined to be C_25_H_24_O_11_, indicating 14 degrees of unsaturation. The IR spectrum showed the presence of hydroxyl (3535 cm^−1^), carbonyl (1668 cm^−1^) and benzene ring (1606, 1506 cm^−1^) groups. The structure of the compound was established from detailed analysis of its ^1^H- and ^13^C-NMR spectra, including 2D NMR. The ^13^C-NMR spectrum of **1** (Table 1), exhibited 25 signals that together with the information from a DEPT spectrum, indicated two methylene, fourteen methine, and nine quaternary carbons. Among these were one carbonyl group at *δ* 196.1 (C-9) and one ester function at *δ* 166.1 (C-19); as well as one anomeric methine at *δ* 100.7 (C-1') and four methines at *δ* 72.7 (C-2'), 75.5 (C-3'), 70.6 (C-4') and 73.7 (C-5'), and one methylene at *δ* 65.2 (C-6'), indicating the presence of a glucopyranose unit. Acid hydrolysis of **1** released D-glucopyranose, with an 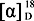 +38.2 (*c* 0.15, H_2_O). The glucopyranose moiety was determined to have a *β*-configuration at C-1' from the large coupling constant of H-1' (*J* = 7.8 Hz) [2]. The ^1^H-NMR spectrum (Table 1) displayed signals for an ABX spin system at *δ* 7.40 (1H, d, *J* = 1.8 Hz, H-2), 7.08 (1H, dd, *J* = 8.4, 1.8 Hz, H-6) and 6.80 (1H, d, *J* = 8.4 Hz, H-5); two *trans*-configured double bonds at *δ* 7.33 (1H, d, *J* = 15.6 Hz, H-7) and *δ* 6.90 (1H, d, *J* = 15.6 Hz, H-8), *δ* 8.02 (1H, d, *J* = 15.6 Hz, H-17) and *δ* 6.10 (1H, d, *J* = 15.6 Hz, H-18) which were further confirmed by the HMQC correlations (H-7/C-7, H-8/C-8, H-17/C-17 and H-18/C-18) and ^1^H-^1^H COSY (Figure 2) correlations (H-7/H-8 and H-17/H-18); two singlet signals at *δ* 7.00 (1H, s, H-13) and *δ* 6.75 (1H, s, H-16) which suggests the presence of an 1,2,4,5-tetrasubstituted aromatic ring; one methylene group signals at *δ* 3.38 (1H, m, H-10a) and *δ* 4.26 (1H, m, H-10b) showed a correlation with *δ* 44.3 (C-10) in the HMQC. In addition to the above signals, a sugar moiety was identified due to the distinct anomeric signal at *δ* 4.88 (1H, d, *J* = 7.8 Hz, H-1'), four oxymethine protons signals between *δ* 4.10 and *δ* 3.38 and two oxymethylene protons signals at *δ* 4.38 and *δ* 4.29, which were further confirmed by the HMQC correlations and ^1^H-^1^H COSY correlations (Figure 2). Since the above-mentioned groups accounted for 13 degrees of unsaturation, the remaining degree suggested the presence of an additional ring system in the structure of **1**. The HMBC experiment (Figure 2) showed clear correlations of ABX protons H-2 and H-6 with C-7, H-7 with C-2 and C-6 and of H-8 with C-1, which confirmed the presence of the carbon-carbon double bond attached at C-1 of the ABX system, while correlations of H-7 with C-9 and of H-8 with C-9 and C-10 confirmed the presence of the carbonyl group C-9 attached at C-8. Correlations of H-10a and H-10b with C-9, C-12 (*δ* 123.4) and C-16 (*δ* 117.9), and of H-16 with C-10 confirmed the attachment of C-10 at carbonyl group (C-9) and C-11 of the tetrasubstituted aromatic ring at C-10.

**Table 1:** ^1^H-NMR (600 MHz) and ^13^C-NMR (150 MHz) data for compound **1** (DMSO-*d*_6_, *δ*_H_ in ppm, *J* in Hz).

Position	δ H	δ C
1	-	125.7
2	7.40 (d, 1.8)	112.4
3	-	145.6
4	-	149.4
5	6.80 (d, 8.4)	116.0
6	7.08 (dd, 8.4, 1.8)	126.4
7	7.33 (d, 15.6)	143.2
8	6.90 (d, 15.6*)*	122.3
9	-	196.1
10a	3.38 (m)	44.3
10b	4.26 (m)	
11	-	127.5
12	-	123.4
13	7.00 (s)	113.2
14	-	144.8
15	-	148.3
16	6.75 (s)	117.9
17	8.02 (d, 15.6)	140.9
18	6.10 (d, 15.6)	116.4
19	-	166.1
1'	4.88 (d, 7.8)	100.7
2'	3.38 (m)	72.7
3'	3.38 (m)	75.5
4'	3.15 (m)	70.6
5'	4.10 (t, 9.4)	73.7
6'a	4.38 (d, 11.4)	65.2
6'b	4.29 (m)	

Another carbon-carbon double bond attached at C-12 was deduced from correlations of H-18 with C-12, H-17 with C-11 (*δ* 127.5) and C-13 (*δ* 113.2) and of H-13 (*δ* 7.00) with C-17 (*δ* 140.9). In addition, correlations of H-17 and H-18 with C-19 suggested the ester function was attached to C-18. The glucopyranose moiety was found to be attached at C-4 as evidenced by the HMBC correlation from H-1' to C-4 (*δ* 149.4). Besides, the glucopyranose group was linked to C-19 through C-6' as determined by the HMBC correlations from H-6' (δ 4.38 and 4.29) to C-19. Based on this combined evidence, structure of compound **1** was confirmed as that of a novel phenolic glycoside that was trivially named gnaphaffine A. A plausible biogenetic pathway has been discussed for compound **1** herein (Scheme 1). Everlastoside L (**4**) was proposed as the precursor of compound **1** via Friedel-Crafts reaction. The common biological intermediate (4-(3',4'-Dihydroxyphenyl)-3-buten-2-one) [21] may convert into everlastoside L (**4**) via saccharification and esterification reactions.

Gnaphaffine B (**2**) was obtained as a pale yellow solid. Its molecular formula was determined as C_30_H_28_O_13_ by its HR-ESI-MS peak at *m/z* 597.1597 [M−H]^−^ (calcd for C_30_H_29_O_13_, 597.1524). IR absorptions suggested the presence of hydroxyl (3455 cm^−1^), carbonyl (1692 cm^−1^) and phenyl groups (1632, 1502 cm^−1^). The ^1^H-NMR spectrum of compound **2** showed three protons at δ 2.70 (dd, *J* = 17.4, 3.0 Hz), 3.26 (dd, *J* = 12.0, 17.4 Hz) and 5.45 (dd, *J* = 12.0, 3.0Hz), typically assigned to H_2_-3 and H-2 of a flavanol skeleton, an AA'XX' spin system at δ 7.27 (2H, d, *J* = 8.4 Hz, H-2', 6') and 6.76 (2H, d, *J* = 8.4 Hz, H-3', 5'), and two *meta*-coupled protons at δ 6.18 (1H, brs, H-6) and 6.13 (1H, dd, *J* = 1.8, 4.8 Hz, H-8) and 5.45 (1H, dd, *J* = 3.0, 12.0 Hz, H-2), 3.26 (1H, dd, *J* = 12.0, 17.4 Hz, H-3a), 2.70 (1H, dd, *J* = 17.4, 3.0 Hz, H-3b), which suggested the presence of a (*S*)-Naringenin skeleton [22,23,24]; a sugar moiety due to the distinct anomeric signal at δ 5.05 (1H, d, 7.8Hz, H-1'') and oxymethine protons in the rang δ 3.21–5.05. The ^13^C-NMR data (Table 2) showed four methines at δ 69.6 (C-4''), 73.9 (C-2''), 76.4 (C-3'') and 77.1 (C-5''), one methylene at δ 63.2 (C-6'') and an anomeric methine at δ 99.3 (C-1''), indicating the presence of a glucopyranose unit.

**Table 2:** ^1^H-NMR (600 MHz) and ^13^C-NMR (150 MHz) data for compound **2** (DMSO-*d*_6_, *δ*_H_ in ppm, *J* in Hz).

Position	δ H	δ C
2	5.45 (1H, dd, 3.0, 12.0)	78.7
3	3.26 (1H, dd, 12.0, 17.4)	42.1
	2.70 (1H, dd, 17.4, 3.0)	
4	-	197.1
5	-	162.7
6	6.18 (1H, brs)	96.3
7	-	163.2
8	6.13 (1H, dd, 1.8, 4.8)	95.5
9	-	165.0
10	-	103.3
1'	-	128.6
2', 6'	7.27 (2H, d, 8.4)	12.4
3', 5'	6.76 (2H, d,8.4)	115.2
4'	-	157.8
1''	5.05 (1H, d, 7.8)	99.3
2''	3.24 (1H, m)	73.79
3''	3.30 (1H, m)	76.4
4''	3.15 (1H, m)	69.6
5''	3.40 (1H, m)	77.1
6''	4.42 (1H, brd, 12.0)	63.2
	4.11 (1H, dd, 18.0,6.6)	
1'''	-	125.5
2'''	7.02 (1H, d, 4.2 )	114.9
3'''	-	145.6
4'''	-	148.4
5'''	6.76 (1H, d, 8.4)	115.7
6'''	6.94 (1H, dd, 8.4,2.4)	121.2
7'''	6.24 (1H, d, 15.6)	113.7
8'''	7.45(1H, d, 15.6)	145.3
9'''	-	166.4

Acid hydrolysis of **2** yielded a free sugar that was identified as D-glucopyranose by measurement of the corresponding optical rotation 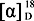 +40.3 (c 0.20, H_2_O). The glucopyranose moiety was determined to have a *β-*configuration at C-1'' from the large coupling constant of H-1'' (*J* = 7.8 Hz) [2]. Furthermore, presence of a caffeoly moiety was confirmed by the detection of ABX spin system signals at 7.02 (1H, d, *J* = 4.2 Hz, H-2'''), 6.76 (1H, d, *J* = 8.4Hz, H-5'''), 6.94 (1H, dd, *J* = 8.4, 2.4 Hz, H-6'''), and the *trans*-configuration of the double bond was recognized by the large coupling constant (*J* = 15.6) observed for the olefinic resonances H-7''' and H-8'''. The glucopyranose attachment at C-7 was supported by the HMBC (Figure 2) correlation of H-1'' (5.05) to C-7 (165.0). The glucopyranose group was linked to C-9''' through C-6''', as confirmed by the HMBC correlation from H-6'' (δ 4.42 and 4.11) to C-9'''. These suggested the glucopyranose group was linked to C-9''' through C-6''. All proton and carbon signals were assigned via HMQC, HMBC (Figure 2) and ^1^H-^1^H COSY spectra. Therefore, compound **2** was identified as naringenin-7-*O-*β-d-(6''-*E*-caffeoyl)-glucopyranoside, and named gnaphaffine B.

Previous studies have already reported on the anti-complementary activity of components from *G. affine* [2,16]. Compounds **1**–**8** were also evaluated *in vitro* for anti-complementary activity on the classical pathway of the complement. Heparin, with an IC_50_ value of 0.016 mg/mL, was used as positive control in this study. Compounds **2**, **3**, **4** and **7** caused moderate inhibtion, showing IC_50_ values of 0.471, 0.221, 0.577 and 1.041 mg/mL, respectively. IC_50_ values for the remaing compounds **1**, **5**, **6** and **8** were 69.63, 81.50, 23.01, and 13.36 mg/mL, respectively.

## 3. Experimental

### 3.1. General

Optical rotations were measured with Perkin-Elmer 341 polarimeter. UV and IR spectra were recorded on Shimadzu UV-2550 and Perkin-Elmer 577 (using KBr disks) spectrophotometers, respectively. NMR spectra were acquired on a Bruker Avance III spectrometer (600 MHz for ^1^H-NMR, 150 MHz for ^13^C-NMR, data in ppm relative to TMS). ESI-MS spectra were recorded on an Agilent 1200 series HPLC interfaced to an Agilent 6410 triple-quadrupole mass spectrometer equipped with an electrospray ionization source, and HR-ESI-MS spectra were recorded on an Agilent 1290 series HPLC interfaced to an Agilent 6538 UHD Accurate-Mass Q-TOF LC/MS (Agilent Corporation, Santa Clara, MA, USA). Semi-preparative RP-HPLC isolation was performed with an Agilent 1200 instrument with a refractive index detector (RID) using a YMC 5 μm C8 column (250 mm × 10 mm). Methanol for semi-preparative HPLC was of HPLC-grade (Merck, Darmstadt, Germany). Column chromatography: silica gel (200−300 mesh); macroporous adsorbing resin (D-101, ZTC-1, 0.3−1.2 mm, Tianjin Zhentiancheng Science & Technology Co., Ltd., Tianjin, China); Sephadex LH-20 gel (40−70 μm, Amersham Pharmacia Biotech AB, Uppsala, Sweden); silica gel H (Qingdao Haiyang Chemical Co. Ltd., Qingdao, China). All solvents for column chromatography and acid hydrolysis were of analytical grade (Shanghai Chemical Reagents Company, Ltd., Shanghai, China). Spots of compounds on TLC were developed using 10% H_2_SO_4_-EtOH solution

### 3.2. Plant Material

The whole *G. affine* plants were purchased at the Bozhou herbal market in Anhui Province, China, in July 2008 and identified by Prof. Wansheng Chen. A voucher specimen was deposited in the Department of Pharmacognosy, Second Military Medical University, Shanghai, China.

### 3.3. Extraction and Isolation

The dried, whole plant materials of *G. affine* (2.7 kg) were ground and extracted with 80% EtOH three times (25 L, each for 2 h) under reflux at 70–80 °C. Evaporation of the solvent at 60 °C yielded a crude extract (338 g), which was suspended in distilled water and successively partitioned with petroleum ether, ethyl acetate, and *n*-butanol. The ethyl acetate extract (60 g) was fractionated by silica gel column chromatography (100–200 mesh, 720 g), using a gradient of CH_2_Cl_2_: MeOH (50:1→1:1; each 5 L, v/v) to yield seven fractions (A→G). Fraction C was chromatographed over a Sephadex LH-20 column eluted with a gradient of MeOH: H_2_O (50:50, 80:20, 0:100, v/v) to give five combined sub-fractions (C_1_‒C_3_). Fraction C_1_ was eluted on RP-18 gel column (MeOH: H_2_O, 3:7, 5:5, 8:2, v/v, each 500ml) to obtain three parts (C_1-1_-C_1-3_). Fraction C_1-1_ was further purified by Sephadex LH-20 CC with MeOH: H_2_O (5:5, 8:2, v/v) to afford compounds **1** (20 mg) and **2** (28 mg). Fraction C_1-2_ was purified on a Sephadex LH-20 column (MeOH: H_2_0, 3:2, v/v) and further subjected to RP-18 gel CC (MeOH:H_2_O, 2:3, v/v) to yield compound **3** (18 mg). Compounds **4** (25 mg) and **5** (18 mg) were obtained from fraction C_1-3_ using the same way as described for compound **3**. Further purification of subfraction C_2_ by RP-18 gel CC (MeOH: H_2_O, 2:3; v/v) and Sephadex LH-20 CC (MeOH: H_2_O, 1:1; v/v) yielded compounds **6** (17 mg) and **7** (20 mg). Further purification of C_3_ by RP-18 gel CC (MeOH: H_2_O, 8:2; v/v) and Sephadex LH-20 CC (CHCl_3_: MeOH, 1:1; v/v) yielded compound **8** (26 mg).

### 3.4. Characterization of Compounds **1** and **2**

Compound **1**. Yellow, amorphous powder; IR (KBr) ν_max_ 3535, 3414, 1668, 1591, 1515, 1288, 1172, 1077 cm^−1^; ^13^C- (150 MHz, DMSO-*d*_6_) and ^1^H-NMR (600 MHz, DMSO-*d*_6_), see Table 1 and Supplementary Data; HR-ESI-MS: *m/z* 501.1393 [M+H]^+^ (calcd for C_25_H_24_O_11_, *m/z* 501.1391).

Compound **2**. Pale yellow solid; IR (KBr) ν_max_ 3856, 3455, 1787, 1692, 1632, 1502, 1274, 1175, 1085 cm^−1^; ^13^C- (150 MHz, DMSO-*d*_6_) and ^1^H-NMR (600 MHz, DMSO-*d*_6_), see Table 2 and Supplementary Data; HR-ESI-MS: *m/z* 597.1597 [M−H]^−^ (calcd for C_30_H_29_O_13_, *m/z* 597.1524).

### 3.5. Acid Hydrolysis of Compounds **1** and **2**

A solution of compound **1** (10 mg) or compound **2** (10 mg) in 2 N aqueous CF_3_COOH (10 mL) was refluxed at 80 °C for 2 h. The mixture was then diluted in water (10 mL) and extracted with EtOAc (3 × 3 mL). The combined EtOAc layers were washed with H_2_O and evaporated to dryness to afford the glycoside. The residue was purified over an ODS column to afford D-glucopyranose (1.2 mg), which was identified on the basis of its specific rotation: 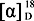 +38.2 (*c* 0.15, H_2_O); 0.8 mg, 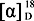 +40.3 (*c* 0.20, H_2_O).

### 3.6. Anti-Complementary Activity Assay

Based on Mayers’ modified method, nornal human serum (NHS) obtained from healthy male donors (mean age 20 years) was used as the complement source, and it was treated with SRBC to remove the anti-sheep erythrocyte antibody. The NHS was diluted 1:10 with veronal-buffered saline (VBS^2+^, PH 7.4, containing 0.5 mM Mg^2+^ and 0.15 mM Ca^2+^) and selected to give submaximallysis in the absence of complement inhibitors. Each saple was dissolved in VBS^2+^ with 1% dimethyl sulfoxide (DMSO). Heparin served as the positive control. The cells culture and biological activity assay were very similar to our previous study [25]. Optical density of the suupernatant was measured at 405nm with a spectrophotometer.The inhition rate of haemolysis was calculated by the following formula: [*A* − (*A*_1_ − *A*_0_)]**/***A* × 100%, where *A* is the absorbance of 100% lysis; *A*_1_ is the absorbance of the sample; and *A*_0_ is the absorbance of control.

## 4. Conclusions

In conclusion, our phytochemical investigation of the roots extract of *G. affine* has identified two new phenolic glycosides, gnaphaffine A (**1)** and B (**2**), together with six known compounds, caffeic acid (**3**), everlastoside L (**4**), isorhamnetin-7-*O*-β-d-glucopyranoside (**5**), quercetin-3-*O*-β-d-glucopyranoside (**6**), scutellarein-7-*O*-β-d-glucopyranoside (**7**) and apigenin-7-*O*-β-d-glucopyranoside (**8**). A plausible biogenetic pathway has been proposed for compound **1** herein (Scheme. 1). The key precursor (4-(3',4'-Dihydroxyphenyl)-3-buten-2-one) may convert into key intermediate everlastoside L (**4**) via saccharification and esterification reactions. Cyclization of the everlastoside L (**4**) yielded compound **1**, which was converted via Friedel-Crafts reaction. Anti-inflammatory activities for compounds **1**–**8** have been evaluated and compounds **2**, **3**, **4** and **7** exhibited significant *in vitro* anti-inflammatory activities through the classic anti-complementary pathway (IC_50_ value < 10 mg/mL).
